# Glucagon-like peptide-1 (GLP-1) mediates the protective effects of dipeptidyl peptidase IV inhibition on pulmonary hypertension

**DOI:** 10.1186/s12929-019-0496-y

**Published:** 2019-01-12

**Authors:** Jingjing Wang, Min Yu, Jian Xu, Yusheng Cheng, Xiang Li, Guihong Wei, Hong Wang, Hui Kong, Weiping Xie

**Affiliations:** 1 Department of Respiratory Medicine, Shanghai Pulmonary Hospital, Tongji University School of Medicine, Shanghai, 200433 People’s Republic of China; 2 Department of Respiratory and Critical Care Medicine, The First Affiliated Hospital of Nanjing Medical University, 300 Guangzhou Road, Nanjing, Jiangsu 210029 People’s Republic of China; 3 Department of Respiratory and Critical Care Medicine, Yijishan Hospital of Wannan Medical College, Wuhu, Anhui 241001 People’s Republic of China

**Keywords:** DPP-4i, GLP-1, Pulmonary vascular remodeling

## Abstract

**Background:**

Pulmonary hypertension (PH) is a progressive disease leading to death ultimately. Our recently published data demonstrated that inhibiting dipeptidyl peptidase IV (DPP-4) alleviated pulmonary vascular remodeling in experimental PH. However, whether glucagon-like peptide-1 (GLP-1) mediated the protective effect of DPP-4 inhibition (DPP-4i) on PH is unclear.

**Results:**

In the present study, GLP-1 receptor antagonist (exendin-3) abolished the protective effects of DPP-4 inhibitor (sitagliptin) on right ventricular systolic pressure (RVSP) and pulmonary vascular remodeling (PVR) in monocrotaline (MCT, 60 mg/kg)-induced PH in rat. Notably, activation of GLP-1 receptor by GLP-1 analogue liraglutide directly attenuated RVSP and PVR in MCT-induced PH, as well as bleomycin- and chronic hypoxia-induced PH. Moreover, liraglutide potently inhibited MCT-induced inflammation and suppressed MCT-induced down-regulation of vascular endothelial marker (VE-cadherin and vWF) in lung. In vitro studies showed liraglutide reversed TGF-β1 (5 ng/ml) combining IL-1β (5 ng/ml) induced endothelial-mesenchymal transition (EndMT) in human umbilical vein endothelial cells (HUVECs), which could be abolished by GLP-1 receptor antagonist (exendin-3). Furtermore, liraglutide suppressed TGF-β1-IL-1β-induced phosphorylation of both Smad3 and ERK1/2.

**Conclusions:**

Our data suggest that GLP-1 mediated the protective effects of DPP-4i on pulmonary vascular and RV remodeling in experimental PH, which may be attributed to the inhibitory effect on EndMT.

## Background

Pulmonary hypertension (PH), characterized by pulmonary vasoconstriction and vascular remodeling, is a progressive disease leading to right ventricular (RV) failure and finally death [1, 2]. The hyperproliferative responses of pulmonary vascular medial smooth muscle cells contribute to the thickening of large elastic vessels and the muscularization of small arteries, thereafter leading to increased pulmonary vascular resistance [3, 4]. Besides, accumulation of inflammatory cells around pulmonary arteries, including macrophages and mast cells, is another obvious profile of pulmonary vascular remodeling (PRV) [5]. Recently, endothelial-mesenchymal transition (EndMT) has been noted in the pulmonary vascular intima both in patients with PH [6, 7] and in animal model of PH [6, 8]. EndMT results in endothelial dysfunction and pro-inflammatory response [9]. Nowadays, inhibiting EndMT has been suggested as a promising therapeutic strategy for the treatment of PH [10].

Our previous study demonstrated that dipeptidyl peptidase IV (DPP-4) inhibition (DPP-4i) alleviated pulmonary vascular remodeling in monocrotaline (MCT)-induced PH rats [11]. DPP-4 is a serine protease which selectively cleaves off N-terminal dipeptides from its substrates such as glucagon-like peptide-1 (GLP-1) [12]. GLP-1, an incretin hormone derived from enteroendocrine cells, can be rapidly degraded by DPP-4, resulting in a short half-life time about 2 min [13]. Accumulating data from both pre-clinical and clinical studies suggest that GLP-1 or GLP-1 analogues are beneficial for cardiovascular diseases (CVDs). For instance, infusion of recombinant GLP-1 could improve LV function in animals with advanced dilated cardiomyopathy [14]. Activating GLP-1 receptor (GLP-1R) with GLP-1 analogue could improve the survival of human aortic endothelial cells after ischemia-reperfusion injury [15]. Notably, GLP-1 could induce an endothelial-dependent relaxation of norepinephrine-challenged constriction of pulmonary artery rings [16]. These results suggest activation of GLP-1/GLP-1R axis may be beneficial for pulmonary circulation under specific stress conditions. However, whether GLP-1/GLP-1R axis could mediate the therapeutic effect of DPP-4i on PH or could exert anti-PH activity itself needs to be further validated. In the present study, sitagliptin (a selective DPP-4 inhibitor), exendin-3 (a GLP-1R antagonist), and liraglutide (a GLP-1 analogue) were used to investigate the effect of GLP-1/GLP-1R axis on PRV in rat model of MCT-induced PH. In addition, the effects of GLP-1R activation on endothelial-mesenchymal transition (EndMT) of human umbilical vein endothelial cells (HUVECs) were investigated.

## Materials and methods

### Ethical approval

All procedures in the present study were performed according to the National Institutes of Health Guide for the Care and Use of Laboratory Animals (publication no. 85–23, revised 1996) and approved by the Institutional Animal Care and Use Committee of Nanjing Medical University (NJMU/IACUC-1601196).

### Experimental animals and design

For MCT model of PH, 64 male Sprague-Dawley (SD) rats (200 ± 10 g) (Shanghai Bikai Laboratory Animal Company, Shanghai, China) were randomly assigned into two groups after raised under standard laboratory conditions for 1 week. One group received an intraperitoneal injection of 0.8-mL saline (*n* = 24), when the other group was injected intraperitoneally with MCT (60 mg/kg, Sigma-Aldrich, MO, USA). Once rats were challenged with MCT, 16 of them were administrated with sitagliptin (SG) daily (80 mg/kg, gavage, Januvia, Merck Sharp, UK) with/without exendin-3 injection (Ex-3, 40 μg/kg, intraperitoneal, Santa Cruz Biotechnology, USA). Eight MCT-treated rats and eight saline-treated rats were randomly picked up for a daily subcutaneous injection with liraglutide (Li) at the doses of 0.2 mg/kg (NovoNordiskA/S, Denmark). The body weights of the rats were measured every other 2 days for dose adjustment during the following 4 weeks.

For bleomycin (BLM) model of PH, 32 rats were assigned into two groups randomly. One group (*n* = 16) was treated with 50-μL 0.9% saline intratracheally, when the other group (*n* = 16) was given an intratracheal administration of BLM (4 U/kg, Nippon Kayaku, Tokyo, Japan). Thereafter on the same day, half of the rats in two groups were daily injected with liraglutide (0.2 mg/kg, subcutaneous) for 4 weeks.

For chronic hypoxia model of PH, 16 rats were housed under normal oxygen, when the other 16 rats were raised in a hypoxia chamber (Biospherix Ltd., USA) for 4 weeks in which the fraction of oxygen was maintained at 10%. Eight rats out of these two groups (normoxia and hypoxia) were injected with liraglutide daily (0.2 mg/kg, subcutaneous), when the rest rats were given 0.2 mL saline daily.

### Hemodynamic measurement and specimen collection

On day 28, after rats were anesthetized by 1% pentobarbital sodium (60 mg/kg, intraperitoneal), RV systolic pressure (RVSP) was determined using right-heart catheterization via right jugular vein according to the protocol described previously [17]. Thereafter, rats were sacrificed for specimen collection. The lung and RV tissues were harvested for morphological evaluation, quantitative real-time reverse transcriptase polymerase chain reaction (RT-qPCR), and Western blot analysis. The weights of RV as well as left ventricle + interventricular septum (LV + S) were measured respectively.

### Assessment of pulmonary morphology

Collected rat lung tissues and RV tissues were immediately fixed in 4% polyformaldehyde (pH 7.4) for 24 h, embedded in paraffin, and then sliced into 4 μm sections. Then lung sections were stained with hematoxylin and eosin (H&E), toluidine blue (TB), picrosirius red (PSR) staining, Masson trichrome staining (MTS), and primary antibody against CD68 (Abcam, MA, USA) according to the protocols described previously [17]. Pulmonary arterial wall thickness (PAWT) was determined according to the ratio of (external diameter − internal diameter) to external diameter based on H&E staining. The right ventricular hypertrophy was determined by the cross section area (CSA) of cardiomyocytes using H&E staining. Graphs were observed under a Leica 2500 microscope (Leica Microsystems, Wetzlar, Germany).

### Cell culture

Human umbilical vein endothelial cells (HUVECs), purchased from ScienCell (CA, USA), were maintained in endothelial growth medium (EGM, ScienCell) containing penicillin (10 U/mL), streptomycin (10 μg/mL), and 5% heat-inactivated fetal bovine serum (ScienCell) as well as endothelial growth factors. When cells (passage 6–7) grew to 100% confluence, EGM was replaced by basal medium with 0.5% serum. Eight hours later, cells were challenged using TGF-β1 (5 μg/ml) & IL-1β (5 μg/ml) with/without liraglutide (25, 50, 100 nM) or exendin 3 (200 nM) pretreatment for 30 min at indicated concentrations. For detecting intercellular signaling transduction, cells were collected for protein extraction and Western blot analysis after challenged with TGF-β1 & IL-1β for 1 h, while for other detections, cells were harvested 72 h after TGF-β1 & IL-1β challenge.

### Identification of EndMT

After cells were challenged with TGF-β1-IL-1β for 72 h, cells were observed under a phase contrast microscope (Nikon, Tokyo, Japan), then fixed in 4% polyformaldehyde for 30 min, blocked with 3% FBS, incubated with primary antibodies against vascular endothelial cadherin (VE-cadherin) and vimentin overnight at 4 °C, and then labeled with second antibody (donkey anti-mouse IgG, Alexa Fluor 555; and donkey anti-rat IgG, Alexa Fluor 488) at room temperature for 1 h. The morphologies of HUVECs were then identified under a Leica 2500 microscope. Besides, cells were also collected for western blot analysis to determine the expression of endothelial markers [VE-cadherin, zonula occludens-1 (ZO-1)] and mesenchymal markers [alpha smooth muscle Actin (α-SMA) and Vimentin].

### Western blot and RT-qPCR

As described previously [18], total protein extracted from lung tissues and HUVECs were electrophoresed on a 8 or 10% sodium dodecyl sulfate (SDS)-polyacrylamide gel, electroblotted to a polyvinylidene difluoride membrane (Millipore, Billerica, MA), incubated with primary antibodies against DPP-4 (Abcam), GLP-1 (Abcam), VE-cadherin (Abcam), Fibronectin (Abcam), α-SMA (Abcam), ZO-1 (ProteinTech Group, Inc., Chicago, IL, USA), vimentin, Smad2/3, p-Smad2/3, extracellular regulated protein kinases1/2 (ERK1/2), p-ERK1/2 (Cell Signaling Technology, MA, USA), or β-actin (ProteinTech Group). After 18 h, the membrane was incubated with HRP-conjugated second antibody for 1 h at room temperature, and then labeled with chemiluminescence (ECL, Cell Signaling Technology). Band signal intensities were analyzed densitometrically using ImageJ software.

Total RNA was extracted from lung tissues using Trizol reagent (Gibco BRL, Grand Island, NY) according to the manufacturer’s instructions. Reverse transcription was conducted with SYBR Premix Ex Taq™ kit (TaKaRa Bio Inc., Dalian, China). Quantitative RCR was performed using an ABI Prism 7500 FAST apparatus (Applied Biosystems, Foster City, CA, USA). The expression for each gene was determined as the relative expression of the housekeeping gene β-actin. PCR primers are listed in Table 1.

**Table 1:** The primer sequences of targeted RNA

Gene primer	Species		Sequence (5′ to 3′)
tnf-α	Rat	Forward	GCATGATCCGAGATGTGGAA
		Reverse	AGACAGAAGAGCGTGGTGGC
il-1β	Rat	Forward	ACAAGGAGAGACAAGCAACGACAA
		Reverse	TTTCCATCTTCTTCTTTGGGTATTG
il-6	Rat	Forward	AGACTTCACAGAGGATACCACCCAC
		Reverse	CAATCAGAATTGCCATTGCACAA
tgf-β1	Rat	Forward	ATTCCTGGCGTTACCTTG
		Reverse	CCCTGTATTCCGTCTCCT
vimentin	Rat	Forward	GCAAAGCAGGAGTCAAACGAATA
		Reverse	TAGCAGCTTCAAGGGCAAAATTC
fibronectin	Rat	Forward	GAGGCACAAGGTCCGAGAAGAG
		Reverse	GAAACCGTGTAAGGGTCAAAGCA
ve-cadherin	Rat	Forward	GCCAATACTTCCGAATAACCAAAC
		Reverse	TGTCATTCTCATCCAAAACTTCAA
vwf	Rat	Forward	CCAGACACTTGCGTTCATC
		Reverse	CTACGGCTTGCACTATTCA
β-actin	Rat	Forward	CTGAACCCTAAGGCCAACCG
		Reverse	GACCAGAGGCATACAGGGACAA

tnf-α, tumor necrosis factor-α; il-1β, interleukin-1β; il-6, interleukin-6; tgf-β1, transforming growth factor-β1; ve-cadherin, vascular endothelial cadherin; vwf, von Willebrand Factor

### Statistical analysis

All values are presented as means ± S.E.M.. Comparisons among groups were performed using one-way ANOVA followed by least significant difference (LSD) post hoc test. *P* < 0.05 was considered statistically significant.

## Results

### GLP-1R antagonist abolished the protective effects of DPP-4i on PH

**Fig. 1:**
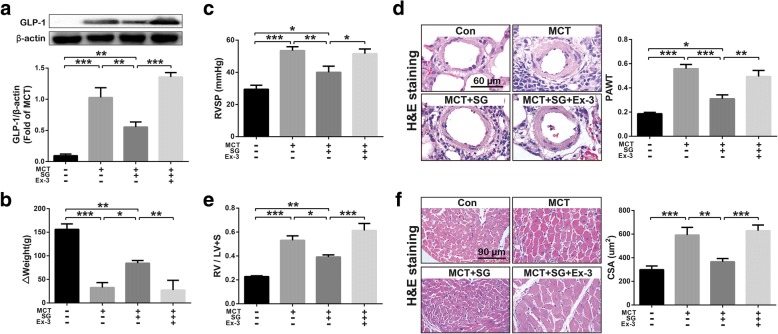
GLP-1R antagonist exendin-3 (Ex-3) abolished the effect of DPP-4i sitagliptin (SG) on GLP-1 expression in lung tissues (**a**), right ventricular systolic pressure (RVSP) (**b**), pulmonary arterial wall thickness (PAWT) (**c**), weight gain during the 4 weeks (ΔWeight) (**d**), and on the ratio of RV to LV + S (**e**), as well as on the hypertrophy of cardiomyocytes [cross section area (CSA) of cardiomyocytes] (**f**). Data were presented as the mean ± s.e.m. (*n* = 6–8). *P < 0.05, **P < 0.01, ***P < 0.001. DPP-4i, Dipeptidyl peptidase IV inhibitor; GLP-1, glucagon-like peptide-1; GLP-1R, GLP-1 receptor; RV, right ventricle; LV + S, left ventricle + interventricular septum

As presented in Fig. 1a, the expression of GLP-1 was almost undetectable in lungs of control rats, while it was significantly expressed in lungs of MCT-challenged rats. Daily treated with DPP-4i (sitagliptin, SG, 80 mg/kg) significantly inhibited MCT-induced up-regulation of GLP-1 in lung tissues. Nevertheless, this effect of SG was completely blocked by the GLP-1R antagonist (Ex-3, 40 μg/kg, daily). Similarly, SG alleviated MCT challenge resulted loss of weight gain (Δweight) in rats, which was alleviated by Ex-3 (Fig. 1b). In addition, the inhibitory effects of SG on MCT-induced increment of RVSP (Fig. 1c), hypertrophy of pulmonary vascular medial layer (Fig. 1d), hypertrophy of RV [the ratio of RV to (LV + S)] (Fig. 1e), and hypertrophy of cardiomyocytes (CSA) (Fig. 1f) were abolished by Ex-3 (Fig. 1c-f). These results indicate that antagonism of GLP-1R could abolish the protective effects of DPP-4i on PH.

### GLP-1 analogue liraglutide alleviated MCT-induced PH

**Fig. 2:**
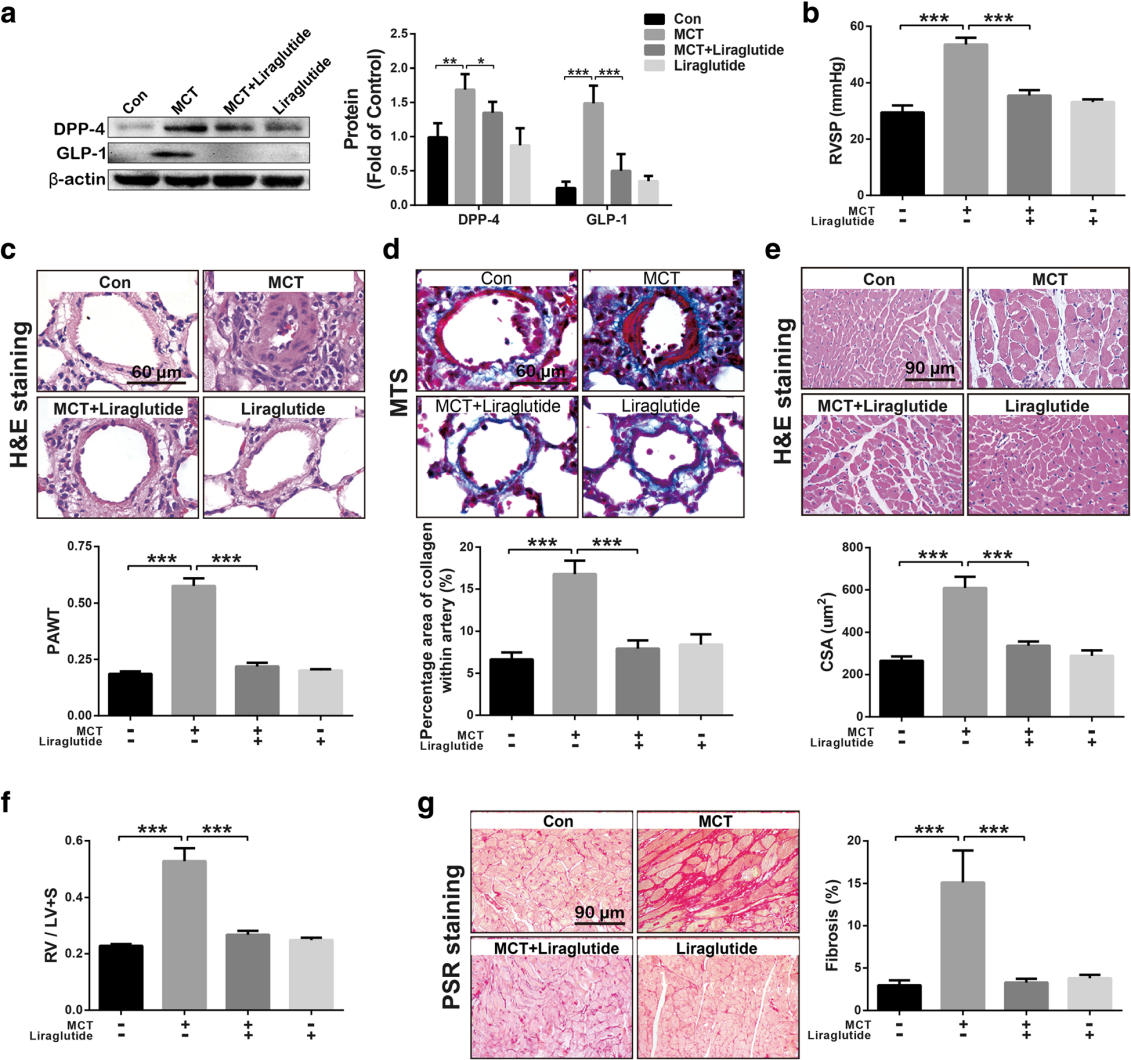
GLP-1 analogue liraglutide inhibited MCT-induced up-regulation in GLP-1 and DPP-4 expression in lung tissues (**a**), reversed the increase in right ventricular systolic pressure (RVSP) (**b**), meanwhile reduces hypertrophy of pulmonary vascular media layer (PAWT) (**c**), pulmonary vascular fibrosis labeled by Masson trichrome staining (MTS) (**d**), cross section area (CSA) of cardiomyocytes (**e**), right ventricle hypertrophy (RV / LV + S) (**f**), and RV fibrosis labeled by picrosirius red staining (PSR) (**g**). Data were presented as the mean ± s.e.m. (*n* = 6–8). *P < 0.05, **P < 0.01, ***P < 0.001

MCT challenge significantly increased DPP-4 and GLP-1 levels in lung tissues, which was inhibited by GLP-1 analogue liraglutide (Fig. 2a). As shown in Fig. 2b, liraglutide significantly reduced MCT challenge-resulted increment of RVSP in rats. Consistently, liraglutide also reversed MCT-induced PVR (Fig. 2c), pulmonary vascular fibrosis (Fig. 2d), cardiomyocyte hypertrophy (Fig. 2e), RV hypertrophy (Fig. 2f), as well as RV fibrosis (Fig. 2g). These results suggest that GLP-1 analogue could prevent MCT-induced PH in rats directly.

### Liraglutide suppressed MCT-induced inflammation and EndMT in pulmonary arteries

**Fig. 3:**
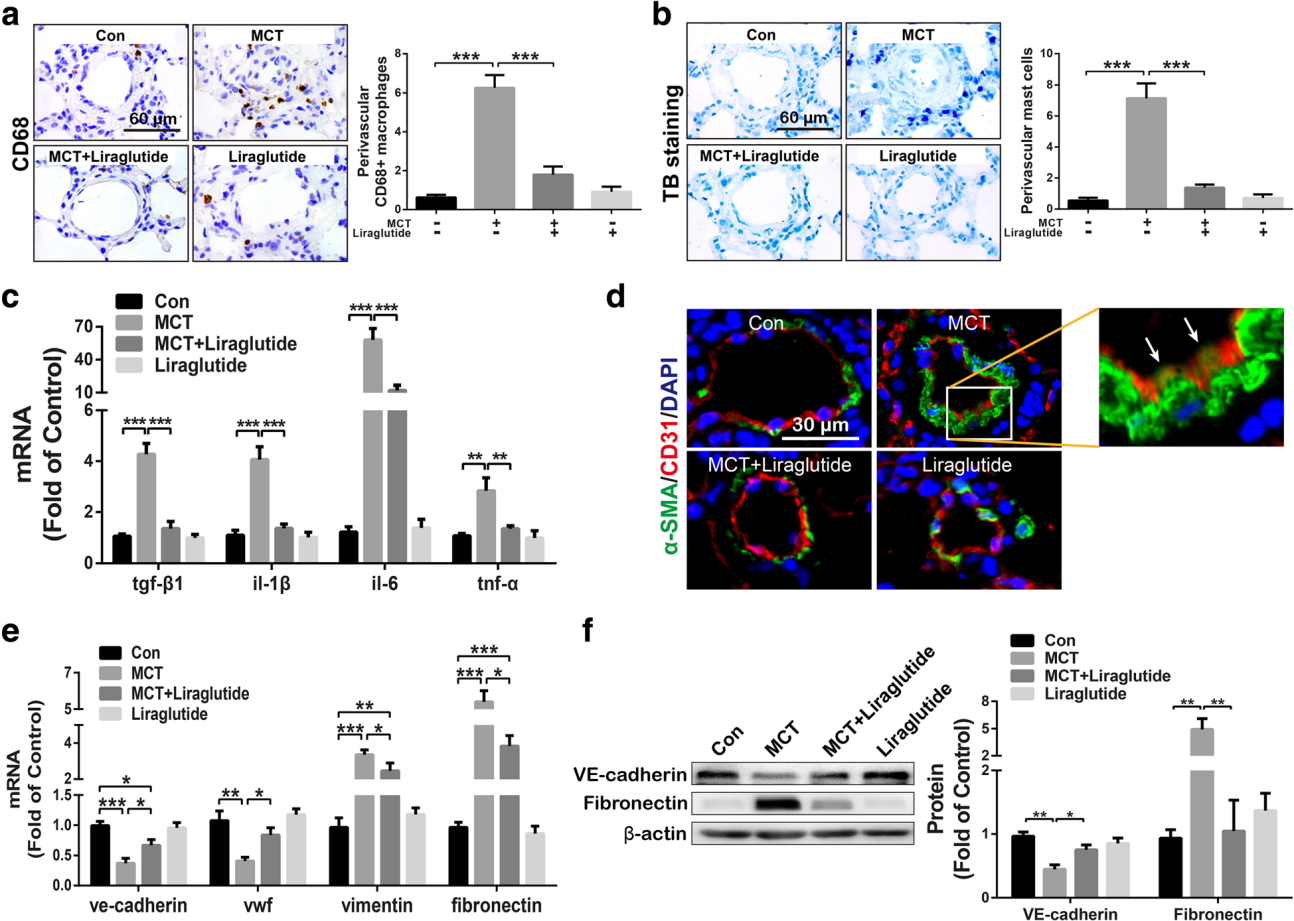
GLP-1 analogue liraglutide suppressed CD68 positive macrophages infiltration (**a**), TB staining positive mast cells accumulation (**b**), and decreased the mRNA expression of tgf-β1, il-1β, il-6 and tnf-α in lung tissues from rats with MCT-induced PH (**c**). Besides, liraglutide reduced the number of α-SMA positive endothelial cells (labeled by CD31) (white arrow) (**d**), and partly recovered the expression of endothelial markers (ve-cadherin and vwf), as well as inhibited mesenchymal markers (vimentin and fibronectin) in mRNA levels in lung tissues from rats with MCT-induced PH (**e**). Furthermore, liraglutide successfully suppressed MCT-induced decrease in VE-cadherin and increase in Fibronectin (**f**). Data were presented as the mean ± s.e.m. (*n* = 6–8). *P < 0.05, **P < 0.01, ***P < 0.001. VE-cadherin, vascular endothelial cadherin; vwf, von Willebrand Factor

Inflammatory cell infiltration around pulmonary vascular contributes to PRV during the development of PH [5]. The accumulation of CD68 positive macrophages around pulmonary arteries was noted in MCT-challenged rats, which was attenuated by the treatment of liraglutide (Fig. 3a). Similarly, liraglutide treatment significantly inhibited the accumulation of TB staining positive mast cells around pulmonary arteries (Fig. 3b). Besides, MCT treatment significantly up-regulated mRNA levels of TGF-β1, IL-1β, IL-6 and TNF-α in lung tissue (Fig. 3c). Consistent with the results from inflammatory cell infiltration in lung, liraglutide completely reversed up-regulation of TGF-β1, IL-1β, TNF-α, and partially reversed up-regulation of IL-6 mRNA in lung induced by MCT treatment.

In addition, immunofluorence analysis showed liraglutide significantly reduced the number of α-SMA positive endothelial cells (labeled by CD31) (Fig. 3d), white arrow). RT-qPCR detection indicated that liraglutide partly reversed MCT-induced down-regulation of endothelial marker mRNA (VE-cadherin and vWF) and up-regulation of mesenchymal marker mRNA (vimentin and fibronectin) in lung (Fig. 3e). In addition, endothelial marker VE-cadherin along with mesenchymal marker fibronectin were selected for further examination by western blot. As indicated in Fig. 3f, liraglutide significantly reversed MCT-induced increase in fibronectin and recovered VE-cadherin partially.

### Liraglutide alleviated BLM- and chronic hypoxia-induced PH

**Fig. 4:**
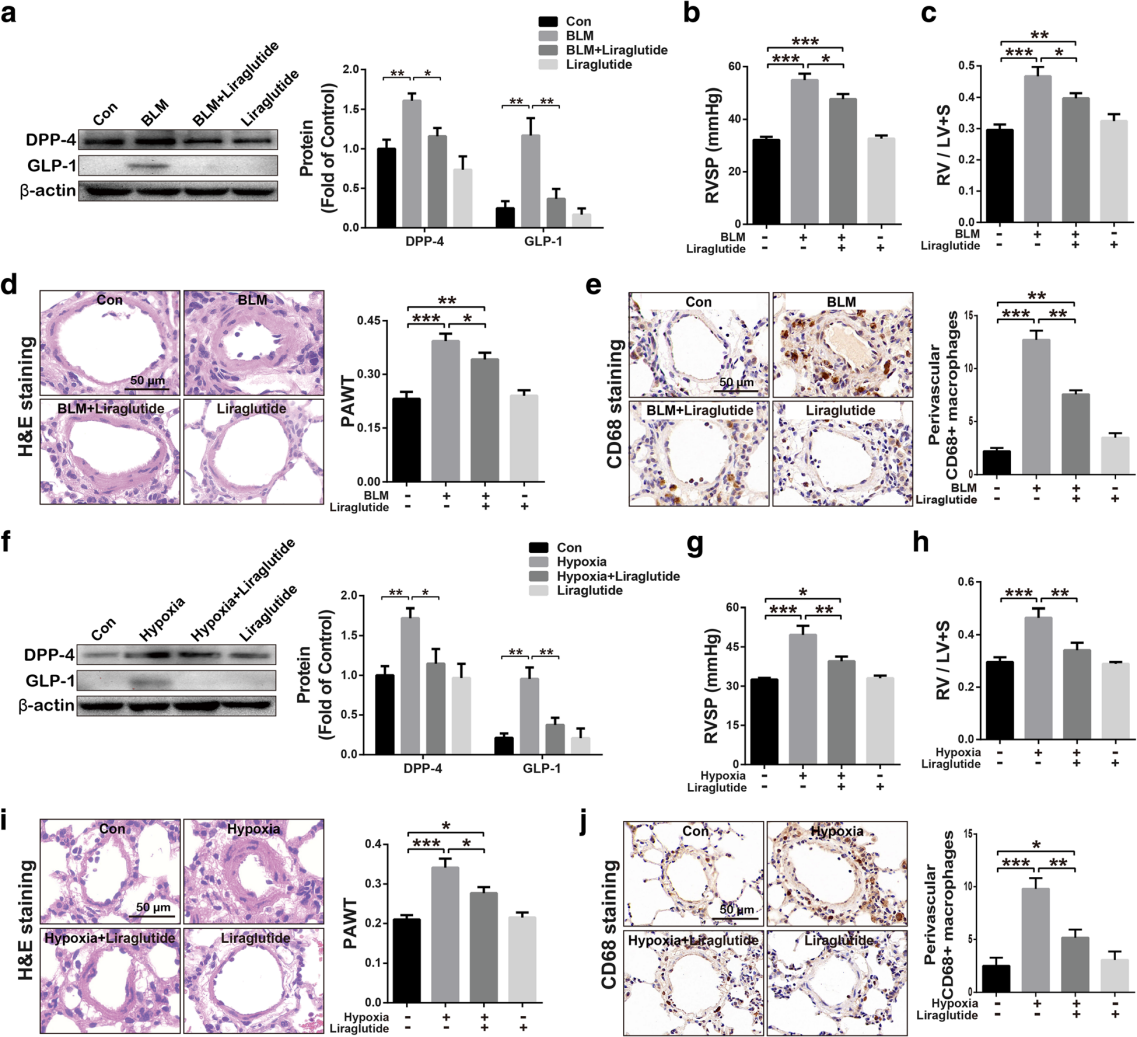
GLP-1 analogue liraglutide partly alleviated bleomycin hydrochloride- and hypoxia-induced PH, including reversing GLP-1 and DPP-4 expression (**a** and **f**), reducing RVSP (**b** and **g**), attenuating RV hypertrophy (RV / LV + S) (**c** and **h**), decreasing the thickness of pulmonary vascular media layer (PAWT) (**d** and **i**), as well as reducing the CD68 positive macrophages infiltration (**e** and **j**). Data were presented as the mean ± s.e.m. (*n* = 6–8). *P < 0.05, **P < 0.01, ***P < 0.001. BLM, bleomycin hydrochloride

BLM-induced PH in rats was established using an intratracheal administration of BLM while chronic hypoxia-related PH was induced through raising rats in a hypoxia chamber (10% oxygen) for 4 weeks. In BLM-PH model, evaluated DPP-4 and GLP-1 were noted in PH rats, which were significantly inhibited by liraglutide treatment (Fig. 4a). Besides, liraglutide significantly alleviated BLM treatment-induced increasing of RVSP (Fig. 4b), hypertrophy of right ventricle (RV/LV + S) (Fig. 4c), thickening of pulmonary vascular medial layer (PAWT) (Fig. 4d), and, accumulation of CD68 positive macrophages (Fig. 4e). In chronic hypoxia-induced PH rats, liraglutide also significantly suppressed hypoxia-induced increase in DPP-4 and GLP-1 expression (Fig. 4f). In addition, liraglutide treatment showed significant beneficial effects as indicated by lower RVSP, RV/LV + S, PAWT, and macrophages infiltration (Fig. 4g-j) compared to chronic hypoxia-challenged rats.

### Liraglutide attenuated TGF-β1 combining IL-1β induced EndMT

**Fig. 5:**
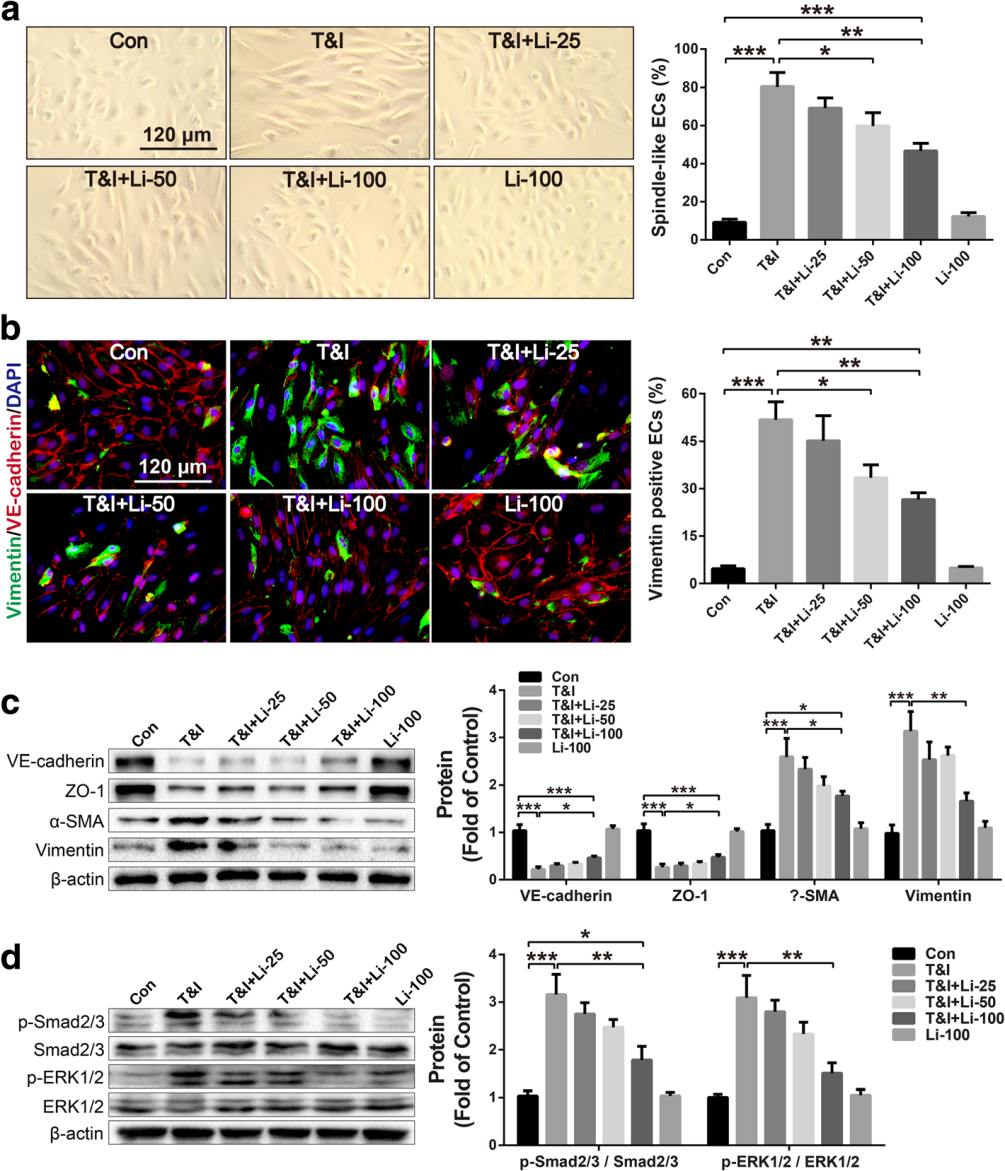
GLP-1 analogue liraglutide attenuated TGF-β1 combining IL-1β induced increment in the percent of spindle-like cells which underwent EndMT (**a**), reduced vimentin positive endothelial cells (labeled by VE-cadherin) undergoing EndMT (**b**), and recovered endothelial markers (VE-cadherin and ZO-1) as well as inhibited mesenchymal markers (α-SMA and vimentin) (**c**). Besides, liraglutide obviously reduced TGF-β1 combining IL-1β induced phosphorylation of Smad2/3 and ERK1/2 (**d**). Data were presented as the mean ± s.e.m. (*n* = 3–4). *P < 0.05, **P < 0.01, ***P < 0.001. T&I, TGF-β1 (5 ng/ml) combining IL-1β (5 ng/ml); EndMT, endothelial-mesenchymal transition; α-SMA, alpha smooth muscle actin; ZO-1, zonula occludens-1; ERK, extracellular signal-regulated kinases; liraglutide, Li, 25, 50, 100 nM

Recently, EndMT was reported to be involved in the development of PH and suggested to be a promising therapeutic target for the treatment of PH [10]. TGF-β1 and IL-1β are key cytokines contributing to EndMT. Therefore, TGF-β1 (5 ng/ml) in combination with IL-1β (5 ng/ml) (T&I) were used to induce EndMT in cultured HUVECs. As shown in Fig. 5a, HUVECs with cobblestone morphology transited to spindle-like morphology 72 h after T&I challenge. Morphology assay found that liraglutide inhibited T&I-induced EndMT of HUVECs in a dose-dependent manner (25, 50, 100 nM). VE-cadherin and vimentin are specific markers for endothelial cells and mesenchymal cells respectively in EndMT. After T&I challenge for 72 h, VE-cadherin between neighboring HUVECs were almost disappeared, while mesenchymal marker vimentin became abundant (Fig. 5b). When treated with liraglutide together, the percent of vimentin-positive cells undergoing EndMT decreased dose-dependently.

For further confirmation, the expressions of endothelial markers (VE-cadherin and ZO-1) and mesenchymal markers (α-SMA and vimentin) in HUVECs were detected by West blotting. As shown in Fig. 5c, T&I challenge resulted in a significant decrease in endothelial markers (VE-cadherin and ZO-1), and a remarkable increase in mesenchymal markers (α-SMA and vimentin). However, these alterations on EndMT-related cell phenotypic markers induced by T&I were significantly inhibited by liraglutide.

During T&I-induced EndMT, Smad and non-Smad signaling are the most important signaling underlying. Therefore, the phosphorylations of Smad2/3 and ERK1/2, representative kinases of Smad and non-Smad signaling respectively, were determined by Western blotting. As shown in Fig. 5d, treated with T&I for 2 h induced a significant phosphorylations of Smad2/3 and ERK1/2 in HUVECs, which were inhibited by liraglutide in a dose-dependent manner.

### GLP-1 analogue suppressed EndMT via GLP-1R

**Fig. 6:**
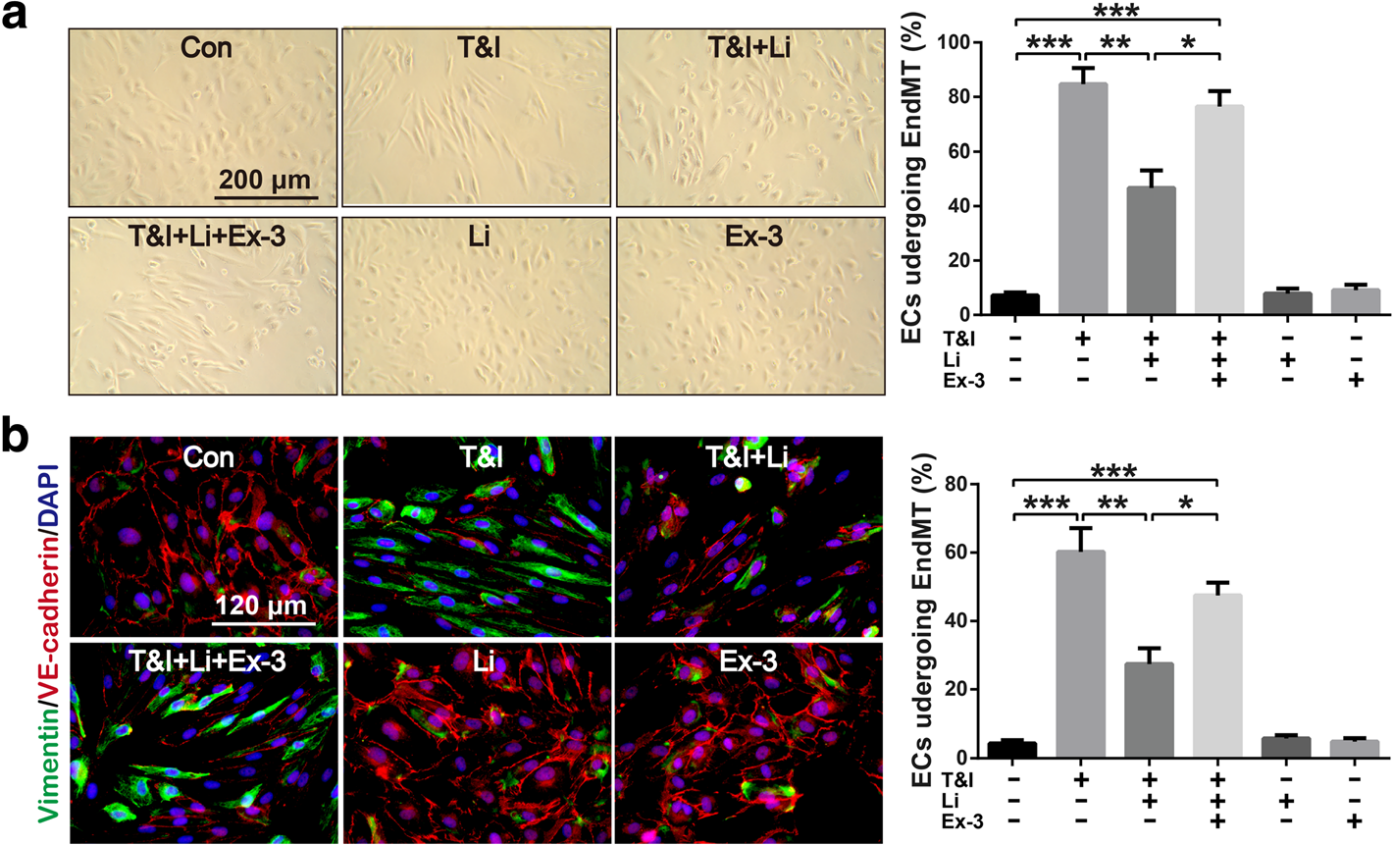
GLP-1 receptor antagonist (exendin-3, 200 nM) abolished the inhibition role of liraglutide in TGF-β1 combining IL-1β induced EndMT, including increase the percent of spindle-like cells (**a**) and vimentin positive endothelial cells (**b**). Data were presented as the mean ± s.e.m. (*n* = 3–4). *P < 0.05, **P < 0.01, ***P < 0.001

When T&I-challenged HUVECs were treated with liraglutide (100 nM), percent of spindle-like cells undergoing EndMT was significantly inhibited (Fig. 6a). However, after liraglutide-treated HUVECs was incubated with GLP-1R antagonist (Ex-3, 200 nM), the percent of spindle-like cells increased obviously. Similarly, liraglutide remarkably suppressed T&I-induced increase in the percent of vimentin+ HUVECs, which was significantly abolished by Ex-3 supplement (Fig. 6b).

## Discussion

In previous study, we reported that DPP-4i suppressed MCT-induced PH in rats [11]. In the current study, we showed that blockage of GLP-1R abolished protective effects of DPP-4i in MCT-induced PH. Moreover, GLP-1 analogue liraglutide successfully alleviated RVSP, pulmonary arterial remodeling, and RV remodeling in rats with MCT-PH, as well as bleomycin- and chronic hypoxia-induced PH. In vitro study on HUVECs provides evidence that GLP-1 analogue could inhibit TGF-β1&IL-1β-challenged activation of Smad/non-Smad signaling, and prevent TGF-β1&IL-1β-induced EndMT.

DPP-4, a serine protease, cleaves a large number of chemokine and peptide hormones and makes them lose biological function [12]. Among them, stromal cell-derived factor-1α (SDF-1α) [19] and brain natriuretic peptide (BNP) [20, 21] presented a protective role in PH. GLP-1 is another important substrate of DPP-4, being inactivated by cleavage of the N-terminal histidine-alanine dipeptide. In the present study, we found GLP-1R antagonist abolished the inhibitory role of DPP-4i in pulmonary vascular remodeling and PH in rats induced by MCT. These results suggested that GLP-1 might be an important downstream molecule mediating the protective effect of DPP-4i on PH. Subsequent study confirmed that GLP-1 could directly prevented pulmonary vascular remodeling in different models of experimental PH. Based on these results, it is suggested that GLP-1 might be a promising target for PH management.

Mounting evidence indicate that GLP-1 exert a broad range of protective effects on cardiovascular diseases, including hypertension [22], chronic heart failure [23, 24], and ischemia-reperfusion injury [25]. Not only in preconstricted systemic circulation arteries, but also in pulmonary arteries, GLP-1 receptor agonist could induce a relaxation in endothelium-dependent manner [16, 26]. In current study, GLP-1 analogue liraglutide alleviates increment of RVSP in different models of PH in rat, suggesting the anti-vasoconstrictive effect may contribute to the therapeutic effect of liraglutide in experimental PH. Besides sustained constriction of pulmonary arteries, pulmonary vascular remodeling characterized by pulmonary arterial intimal and medial hypertrophy is another predominant profile of PH [27].

Inflammatory cytokines (such as TGF-β1 and IL-1β), derived from inflammatory cells, could result in intima EndMT in the development of PH. In the present study, GLP-1 analogue liraglutide treatment successfully attenuated the accumulation of macrophages and mast cells around pulmonary vessels, thereafter inhibited the cytokine release in lung. Till now, the anti-inflammatory effects of GLP-1R agonist have been confirmed in different diseases, such as renal injury [28], atherosclerosis [29], type 2 diabetes mellitus [30], and intracerebral hemorrhage [31].

EndMT, the most recently discovered pathological features of PH, is a complex process in which cells detach from the endothelial layer, lose their specific molecular markers, and acquire a mesenchymal phenotype [32]. It was reported that GLP-1 protected against hyperglycemic-induced EndMT of human aortic endothelial cells and improves myocardial dysfunction [33]. In experimental PH models, EndMT can be induced by chronic hypoxia [34] or inflammatory responses [9]. Our in vivo study showed that endothelial specific markers were significantly down-regulated and mesenchymal markers were up-regulated in lungs of MCT-induced PH rats, while these changes were partially reversed by the treatment of GLP-1 analogue liraglutide. These results suggest that GLP-1R activation may inhibit EndMT in MCT-induced PH. Although we found that GLP-1 analogue significantly inhibit MCT-induced inflammation, the effects of GLP-1R activation on inflammatory cytokines-induced EndMT is unclear. Our in vitro study showed that TGF-β1 combining IL-1β induced EndMT of HUVECs as indicated by the morphologic change and alterations of endothelial/mesenchymal-specific markers. As expected, GLP-1 analogue liraglutide not only inhibited expression of mesenchymal markers (vimentin and α-SMA), but also recovered expression of endothelial markers (VE-cadherin, ZO-1). Therefore, our data suggest that inhibiting EndMT could be one of the potential mechanisms for the therapeutic effects of GLP-1 analogue on PH.

Accumulating evidence has revealed the importance of TGF-β signaling in EndMT [35]. Smad2/3 as well as Smad-independent signaling (including ERK1/2) play an essential role in TGF-β-induced EndMT [36]. In the present study, GLP-1 analogue could reduce the phosphorylation of both Smad2/3 and ERK1/2, suggesting GLP-1 analogue could suppress EndMT by inhibiting Smad-dependent and Smad-independent ways. Consistent with our results, GLP-1 analogue could also reduce the phosphorylation of both Smad2/3 in Ang II-induced cardiac fibrosis, enhance bone morphogenetic proteins (BMP) signaling [37] which in turn inhibited TGF-β/Smad2/3 signaling, and decreased the phosphorylation of ERK1/2 in vascular smooth muscle cells [38, 39].

GLP-1 analogues have also been reported to prevent and reverse monocrotaline-induced pulmonary arterial hypertension by suppressing ET-1 and enhancing eNOS/sGC/PKG pathways [40], and to improve hypoxia-induced pulmonary hypertension in mice partly via normalization of reduced ET(B) receptor expression [41]. Besides, the anti-proliferation effect of GLP-1 analogues on different vascular smooth muscle cells, including pulmonary arterial smooth muscle cells (PASMCs) [40] and aortic smooth muscle cells (ASMCs) [39, 42], might also contribute to the beneficial effect of GLP-1R agonist on PH.

Increased GLP-1 was noted in lung tissues from rats with PH, which means that the rats could partly protect themself against PH via up-regulating GLP-1expresseion. On the other hand, GLP-1, expressed in the lung tissues, might also indicate the severity of PH since GLP-1 was nearly not expressed in lung tissues from control rats. It seems that DPP-4i decreased GLP-1 expression in lung tissues form rats with PH in the present study, but it is not the fact. We suspect that the decreased expression in GLP-1 was mainly attributed to the DPP-4i-mediated alleviation of PH even though DPP-4i could up-regulate GLP-1 directly. Consistent with DPP-4i treatment, GLP-1 analogue liraglutide treatment also reduced GLP-1 expression in lung tissues from rats with PH, which might also result from the alleviation of PH.

## Conclusions

GLP-1 mediated the protective effects of DPP-4i on pulmonary vascular and RV remodeling in experimental PH, which may be attributed to the inhibitory effect on EndMT. Targeted on GLP-1 could be a novel therapeutic option for the treatment of PH.

## Abbreviations

DPP-4: Dipeptidyl peptidase IV
EndMT: Endothelial-mesenchymal transition
GLP-1: Glucagon-like peptide-1
H&E: Hematoxylin and eosin
HUVECs: Human umbilical vein endothelial cells
Li: Liraglutide
MCT: Monocrotaline
MTS: Masson trichrome staining
PH: Pulmonary hypertension
PSR: Picrosirius red staining
PVR: Pulmonary vascular remodeling
RVSP: Right ventricular systolic pressure
SG: Sitagliptin
T&I: TGF-β1 combining IL-1β
TB: Toluidine blue

## References

[1] Lai YC, Potoka KC, Champion HC, Mora AL, Gladwin MT (2014). Pulmonary arterial hypertension: the clinical syndrome. Circ Res.

[2] Thenappan T, Ormiston ML, Ryan JJ, Archer SL (2018). Pulmonary arterial hypertension: pathogenesis and clinical management. BMJ.

[3] Arias-Stella J, Kruger H, Recavarren S (1973). Pathology of chronic mountain sickness. Thorax.

[4] Hunter KS, Lammers SR, Shandas R (2011). Pulmonary vascular stiffness: measurement, modeling, and implications in normal and hypertensive pulmonary circulations. Compr Physiol.

[5] Pugliese SC, Poth JM, Fini MA, Olschewski A, El Kasmi KC, Stenmark KR (2015). The role of inflammation in hypoxic pulmonary hypertension: from cellular mechanisms to clinical phenotypes. Am J Physiol Lung Cell Mol Physiol..

[6] Ranchoux B, Antigny F, Rucker-Martin C, Hautefort A, Pechoux C, Bogaard HJ, Dorfmuller P, Remy S, Lecerf F, Plante S (2015). Endothelial-to-mesenchymal transition in pulmonary hypertension. Circulation.

[7] Jimenez SA, Piera-Velazquez S (2016). Endothelial to mesenchymal transition (EndoMT) in the pathogenesis of systemic sclerosis-associated pulmonary fibrosis and pulmonary arterial hypertension. Myth or reality?. Matrix Biol.

[8] Nikitopoulou I, Orfanos SE, Kotanidou A, Maltabe V, Manitsopoulos N, Karras P, Kouklis P, Armaganidis A, Maniatis NA (2016). Vascular endothelial-cadherin downregulation as a feature of endothelial transdifferentiation in monocrotaline-induced pulmonary hypertension. Am J Physiol Lung Cell Mol Physiol.

[9] Cho JG, Lee A, Chang W, Lee MS, Kim J (2018). Endothelial to mesenchymal transition represents a key link in the interaction between inflammation and endothelial dysfunction. Front Immunol.

[10] Stenmark KR, Frid M, Perros F (2016). Endothelial-to-mesenchymal transition: an evolving paradigm and a promising therapeutic target in PAH. Circulation.

[11] Xu J, Wang J, He M, Han H, Xie W, Wang H, Kong H (2018). Dipeptidyl peptidase IV (DPP-4) inhibition alleviates pulmonary arterial remodeling in experimental pulmonary hypertension. Lab Investig.

[12] Lambeir AM, Durinx C, Scharpe S, De Meester I (2003). Dipeptidyl-peptidase IV from bench to bedside: an update on structural properties, functions, and clinical aspects of the enzyme DPP IV. Crit Rev Clin Lab Sci.

[13] Kieffer TJ, Habener JF (1999). The glucagon-like peptides. Endocr Rev.

[14] Nikolaidis LA, Elahi D, Hentosz T, Doverspike A, Huerbin R, Zourelias L, Stolarski C, Shen YT, Shannon RP (2004). Recombinant glucagon-like peptide-1 increases myocardial glucose uptake and improves left ventricular performance in conscious dogs with pacing-induced dilated cardiomyopathy. Circulation.

[15] Ban K, Kim KH, Cho CK, Sauve M, Diamandis EP, Backx PH, Drucker DJ, Husain M (2010). Glucagon-like peptide (GLP)-1(9-36)amide-mediated cytoprotection is blocked by exendin(9-39) yet does not require the known GLP-1 receptor. Endocrinology.

[16] Golpon HA, Puechner A, Welte T, Wichert PV, Feddersen CO (2001). Vasorelaxant effect of glucagon-like peptide-(7-36) amide and amylin on the pulmonary circulation of the rat. Regul Pept.

[17] Wang JJ, Zuo XR, Xu J, Zhou JY, Kong H, Zeng XN, Xie WP, Cao Q (2016). Evaluation and treatment of endoplasmic reticulum (ER) stress in right ventricular dysfunction during Monocrotaline-induced rat pulmonary arterial hypertension. Cardiovasc Drugs Ther.

[18] Wang J, Xu J, Zhao X, Xie W, Wang H, Kong H (2018). Fasudil inhibits neutrophil-endothelial cell interactions by regulating the expressions of GRP78 and BMPR2. Exp Cell Res.

[19] Yin T, Bader AR, Hou TK, Maron BA, Kao DD, Qian R, Kohane DS, Handy DE, Loscalzo J, Zhang YY (2013). SDF-1alpha in glycan nanoparticles exhibits full activity and reduces pulmonary hypertension in rats. Biomacromolecules.

[20] Hill NS, Klinger JR, Warburton RR, Pietras L, Wrenn DS (1994). Brain natriuretic peptide: possible role in the modulation of hypoxic pulmonary hypertension. Am J Phys.

[21] Klinger JR, Warburton RR, Pietras L, Hill NS (1998). Brain natriuretic peptide inhibits hypoxic pulmonary hypertension in rats. J Appl Physiol (1985).

[22] Sun F, Wu S, Guo S, Yu K, Yang Z, Li L, Zhang Y, Quan X, Ji L, Zhan S (2015). Impact of GLP-1 receptor agonists on blood pressure, heart rate and hypertension among patients with type 2 diabetes: a systematic review and network meta-analysis. Diabetes Res Clin Pract.

[23] Lepore JJ, Olson E, Demopoulos L, Haws T, Fang Z, Barbour AM, Fossler M, Davila-Roman VG, Russell SD, Gropler RJ (2016). Effects of the novel long-acting GLP-1 agonist, Albiglutide, on cardiac function, cardiac metabolism, and exercise capacity in patients with chronic heart failure and reduced ejection fraction. JACC Heart Fail.

[24] Nguyen TD, Shingu Y, Amorim PA, Schenkl C, Schwarzer M, Doenst T (2018). GLP-1 improves diastolic function and survival in heart failure with preserved ejection fraction. J Cardiovasc Transl Res.

[25] Baba S, Iwasa M, Higashi K, Minatoguchi S, Yamada Y, Kanamori H, Kawasaki M, Nishigaki K, Minatoguchi S (2017). Antidiabetic drug Alogliptin protects the heart against ischemia-reperfusion injury through GLP-1 receptor-dependent and receptor-independent pathways involving nitric oxide production in rabbits. J Cardiovasc Pharmacol.

[26] Salheen SM, Panchapakesan U, Pollock CA, Woodman OL (2015). The DPP-4 inhibitor linagliptin and the GLP-1 receptor agonist exendin-4 improve endothelium-dependent relaxation of rat mesenteric arteries in the presence of high glucose. Pharmacol Res.

[27] Bueno-Beti C, Sassi Y, Hajjar RJ, Hadri L (2018). Pulmonary artery hypertension model in rats by Monocrotaline administration. Methods Mol Biol.

[28] Sancar-Bas S, Gezginci-Oktayoglu S, Bolkent S (2015). Exendin-4 attenuates renal tubular injury by decreasing oxidative stress and inflammation in streptozotocin-induced diabetic mice. Growth Factors.

[29] Hirano T, Mori Y (2016). Anti-atherogenic and anti-inflammatory properties of glucagon-like peptide-1, glucose-dependent insulinotropic polypepide, and dipeptidyl peptidase-4 inhibitors in experimental animals. J Diab Investig.

[30] Hogan AE, Gaoatswe G, Lynch L, Corrigan MA, Woods C, O’Connell J, O’Shea D (2014). Glucagon-like peptide 1 analogue therapy directly modulates innate immune-mediated inflammation in individuals with type 2 diabetes mellitus. Diabetologia.

[31] Hou J, Manaenko A, Hakon J, Hansen-Schwartz J, Tang J, Zhang JH (2012). Liraglutide, a long-acting GLP-1 mimetic, and its metabolite attenuate inflammation after intracerebral hemorrhage. J Cereb Blood Flow Metab.

[32] Piera-Velazquez S, Mendoza FA, Jimenez SA. Endothelial to mesenchymal transition (EndoMT) in the pathogenesis of human fibrotic diseases. J Clin Med. 2016;5. 10.3390/jcm5040045 PMC4850468 27077889

[33] Yan F, Zhang GH, Feng M, Zhang W, Zhang JN, Dong WQ, Zhang C, Zhang Y, Chen L, Zhang MX (2015). Glucagon-like peptide 1 protects against hyperglycemic-induced endothelial-to-mesenchymal transition and improves myocardial dysfunction by suppressing poly (ADP-ribose) polymerase 1 activity. Mol Med.

[34] Zhang B, Niu W, Dong HY, Liu ML, Luo Y, Li ZC (2018). Hypoxia induces endothelialmesenchymal transition in pulmonary vascular remodeling. Int J Mol Med.

[35] Van Meeteren LA, Ten Dijke P (2012). Regulation of endothelial cell plasticity by TGF-beta. Cell Tissue Res.

[36] Sanchez-Duffhues G, Garcia De Vinuesa A, Ten Dijke P (2018). Endothelial-to-mesenchymal transition in cardiovascular diseases: developmental signaling pathways gone awry. Dev Dyn.

[37] Yew KH, Hembree M, Prasadan K, Preuett B, Mcfall C, Benjes C, Crowley A, Sharp S, Tulachan S, Mehta S (2005). Cross-talk between bone morphogenetic protein and transforming growth factor-beta signaling is essential for exendin-4-induced insulin-positive differentiation of AR42J cells. J Biol Chem.

[38] Shi L, Ji Y, Jiang X, Zhou L, Xu Y, Li Y, Jiang W, Meng P, Liu X (2015). Liraglutide attenuates high glucose-induced abnormal cell migration, proliferation, and apoptosis of vascular smooth muscle cells by activating the GLP-1 receptor, and inhibiting ERK1/2 and PI3K/Akt signaling pathways. Cardiovasc Diabetol.

[39] Nagayama K, Kyotani Y, Zhao J, Ito S, Ozawa K, Bolstad FA, Yoshizumi M (2015). Exendin-4 prevents vascular smooth muscle cell proliferation and migration by angiotensin II via the inhibition of ERK1/2 and JNK signaling pathways. PLoS One.

[40] Lee MY, Tsai KB, Hsu JH, Shin SJ, Wu JR, Yeh JL (2016). Liraglutide prevents and reverses monocrotaline-induced pulmonary arterial hypertension by suppressing ET-1 and enhancing eNOS/sGC/PKG pathways. Sci Rep.

[41] Honda J, Kimura T, Sakai S, Maruyama H, Tajiri K, Murakoshi N, Homma S, Miyauchi T, Aonuma K (2018). The glucagon-like peptide-1 receptor agonist liraglutide improves hypoxia-induced pulmonary hypertension in mice partly via normalization of reduced ET(B) receptor expression. Physiol Res.

[42] Jojima T, Uchida K, Akimoto K, Tomotsune T, Yanagi K, Iijima T, Suzuki K, Kasai K, Aso Y (2017). Liraglutide, a GLP-1 receptor agonist, inhibits vascular smooth muscle cell proliferation by enhancing AMP-activated protein kinase and cell cycle regulation, and delays atherosclerosis in ApoE deficient mice. Atherosclerosis.

